# Should basic life support–defibrillator training be compulsory for newly licensed Italian physicians? An observational study

**DOI:** 10.2459/JCM.0000000000001645

**Published:** 2024-06-24

**Authors:** Giuseppe Stirparo, Lorenzo Bellini, Daniele Solla, Pierfrancesco Stirparo, Nazzareno Fagoni, Luca Gambolò

**Affiliations:** a SIMED Società Italiana di Medicina e Divulgazione Scientifica, Parma; b Department of Molecular and Translational Medicine, University of Brescia, Brescia, Italy

**Keywords:** basic life support–defibrillator, education, training

## Abstract

**Introduction:**

Out-of-hospital cardiac arrest (OHCA) is a relevant event with a fatal outcome in most cases. Basic life support–defibrillator (BLSD) training is central to rescuing a patient in arrest and ensuring that the patient has a better chance of returning to spontaneous circulation. Despite this, BLSD training is not mandatory for newly licensed physicians. Our study aims to evaluate the preparedness of newly qualified doctors to manage an OHCA and the impact of BLSD training.

**Materials and methods:**

We tested 120 newly qualified doctors, members of the ‘Italian Society of Medicine and Scientific Divulgation’ network, evaluating their practical and theoretical knowledge in managing an OHCA before and after a BLSD training course conducted according to the American Heart Association guidelines.

**Results:**

Fifty-nine physicians (49.2%) had an adequate background of the theoretical basis of cardiopulmonary resuscitation (CPR); 37 (30.8%) were able to perform effective CPR on a mannequin, but only 19 (15.8%) were able to perform effective CPR with adequate depth and frequency of compressions. After the BLSD training course, 111 physicians (92.5%) were able to perform effective and quality CPR on a mannequin with feedback.

**Conclusion:**

In Italy, BLSD training for physicians is not mandatory, and newly licensed physicians showed good knowledge of the theoretical basis of CPR, but few of them performed compressions of adequate depth and frequency. These results should guide future educational policy decisions in Italian academies.

## Introduction

Out-of-hospital cardiac arrest (OHCA) has an average annual incidence of 1 case per 1000, ranging from 28 to 244 per 100 000 worldwide, and represents a significant public health emergency.^1,2^ This emergency, according to scientific literature, is reversible in up to 60% of cases through effective cardiopulmonary resuscitation (CPR) within the first minute of onset, and through early defibrillation with an automated external defibrillator (AED).^3–6^

The importance of effective CPR was also recently emphasized during the COVID-19 pandemic,^7–9^ a period during which there was a reduction in basic life support–defibrillator (BLSD) courses^10^ to increase provider safety.^11^ However, the real impact of reduced BLSD training on OHCA mortality is unclear, although a concomitant increase in OHCA mortality was reported during the COVID-19 pandemic.^12^ Training plays an important role in OHCA management, and the practical BLSD course, according to the leading scientific societies, has a very clear outline. It is a specific training course in which trainees only receive a certificate after demonstrating competence in OHCA management.^13^ The role of training in the proper management of OHCA has been demonstrated by several studies, in both lay people and health professionals.^14–19^

In Italy, participation in BLSD courses is on a voluntary basis for both lay people and healthcare personnel; in fact, for the latter, there is no legal requirement for BLSD training.^20^ Only in workplaces, with Legislative Decree 81/2008, does the law require employers to identify the ‘first-aid responder,’ a worker who is required to undergo compulsory training. Unfortunately, the legislation only requires the course for CPR maneuvers and does not provide any module for AED. Despite this, the literature has shown that there is an improvement in ROSCs in places where there are trained people.^21^ This may seem unusual; in fact, in Italy, academic curricula do not mandatorily include theoretical-practical courses in medical and health profession universities. Because of this, medical students enrolled in their final year may have significant practical deficiencies in emergency management,^22,23^ and yet in many cases they are the ones, newly graduated, who participate in sports or other events where OHCA may occur as their first job.^20,24^

Newly licensed physicians often hold various roles in the Italian National Health System (SSN, Sistema Sanitario Nazionale). Apart from participating in sports competitions as a field physician, they can work in the medical service, as substitutes for general practitioners or work in private clinics. They are an important group to consider for improved CPR education and training, as they are likely to encounter patients with OHCA. However, research on the effectiveness of CPR education programs for this population is limited; in fact, studies focus more on medical students than on newly licensed physicians,^25–27^ who theoretically should already be able to perform effective CPR.

It should also be emphasized that physicians are also citizens, like everyone else, and it would be proper for them to receive BLSD training. In addition, physicians are expected to highlight best practices and be the first to follow them in accordance with scientific evidence, which is why BLSD training and retraining should be done by physicians first, as opposed to citizens in general.

Therefore, this study aims to explore the CPR skills of newly trained physicians. Specifically, we investigated the CPR knowledge and skill levels of newly licensed physicians: by answering these research questions, we hope to contribute to the development of effective CPR education programs for newly licensed physicians and identify strategies to improve bystander CPR rates.

## Materials and methods

This is a retrospective observational study. From October 2019 to December 2020, 120 physicians from a single university, no more than 6 months after graduation, were included in the experiment. We tested the students with a questionnaire according to the final AHA BLS course test (Italian version).

After the theoretical assessment, candidates were asked to perform CPR on a mannequin without feedback for 2 min. Next, candidates were asked to perform CPR on a mannequin with feedback to check if the frequency and depth of chest compressions were adequate.

After the practical evaluation, the candidates were divided into 20 groups of six members each. All groups were administered a specific BLSD training course standardized on AHA guidelines. This course was 8 h in duration and focused on performing CPR on adult patients and defibrillation.

Finally, after the course, candidates were asked to perform CPR on a mannequin connected to a smartphone application for data collection, without looking at the feedback. Only the instructor looked at the visual feedback.

A smartphone application, connected to the mannequins via Bluetooth, was used for visual feedback of resuscitation maneuvers at both training times.

Data on overall performance were collected and analyzed to investigate the impact of the course on CPR practice. Dependency relationships between categorical variables were analyzed by chi-square tests with α = 0.05. The same proportion of sample performance was assessed by McNemar's standard test with α = 0.05.

## Results

The sample of physicians analyzed consisted of 120 newly licensed physicians (not more than 6 months after licensure). Sixty-seven (55.8%) were women. Fifty-nine physicians (49.2%) showed good knowledge of the theoretical basis of CPR, 37 (30.8%) were able to perform CPR on a mannequin, but only 19 (15.8%) were able to perform effective CPR with adequate depth and frequency of compressions.

After the BLSD training course, 111 physicians (92.5%) demonstrated the ability to provide effective and quality CPR on a mannequin with feedback. Table 1 summarizes the general description of the sample.

**Table 1:** Sample description

Population *N* (%)	Theoretical knowledge of CPR *N* (%)	Effective CPR on the mannequin without feedback *N* (%)	Effective CPR on the mannequin with feedback *N* (%)	Effective CPR on the mannequin with feedback after 8 h BLSD training course *N* (%)
Women 67 (55.8%)	29 (43.3%)	17 (25.43)	8 (11.9%)	61 (91.0%)
Men 53 (44.1%)	30 (56.6%)	20 (37.7%)	11 (20.8%)	50 (94.3%)
Total 120 (100%)	59 (49.2%)	37 (30.8%)	19 (15.8%)	111 (92.5%)

The relationship between sex and knowledge/practical ability to perform CPR was analyzed. The results are summarized in Table 2. Chi-square test showed no dependence relationship between the categorical variable ‘Sex’ and the categorical variable ‘Theoretical knowledge of CPR,’ χ^2^ (1, *N* = 120) = 2.1, *P* > 0.05. The categorical variable ‘Effective CPR on the mannequin without feedback’ shows no dependence relationship with the categorical variable ‘Sex,’ χ^2^ (1, *N* = 120) = 2.1, *P* > 0.05. The categorical variable ‘Effective CPR on the mannequin with feedback’ shows no dependence relationship with the categorical variable ‘Sex’, χ^2^ (1, *N* = 120) = 1.7, *P* > 0.05. The categorical variable ‘Effective CPR on the mannequin with feedback after 8 h BLSD training course’ shows no dependence relationship with the categorical variable ‘Sex,’ χ^2^ (1, *N* = 120) = 0.4, *P* > 0.05.

**Table 2:** Relationship between sex and outcomes of interest

	Theoretical knowledge of CPR	Effective CPR on the mannequin without feedback	Effective CPR on the mannequin with feedback	Effective CPR on the mannequin with feedback after 8 h BLSD education course
Sex M/F	χ^2^ = 2.1 *P *> 0.05	χ^2^ = 2.1 *P *> 0.05	χ^2^ = 1.7 *P *> 0.05	χ^2^ = 0.4 *P *> 0.05

Finally, the specific BLSD training course was shown to be effective in enhancing the quality of CPR performance in students. A standard McNemar test determined a statistically significant difference in the percentage of performance in young physicians pre and post-BLSD course intervention (92.5 vs. 15.8%, *P* < 0.05).

## Discussion

This study provides findings regarding the disparity between the percentage of physicians with theoretical CPR knowledge, which reaches 49.2% in our sample, and the percentage of physicians with practical CPR skills, which is even lower at an alarming 15.8%. This is a relevant finding, as the percentage of physicians who can perform quality and effective CPR for 2 minutes is too low. There is a significant gap in the practical ability of newly licensed physicians to perform effective CPR,^23,28^ with only a small percentage of participants being able to perform it with appropriate depth and frequency of compressions. Medical students and physicians emphasize the need for emergency training, to be effective and up to date with the latest scientific findings and technological advances.^29,30^ The proposal for compulsory practical training for medical students is debated in the scientific community; several scientific societies^22,23,28^ have commented on this topic. Scientific research supports the role of the internship in the undergraduate curriculum, which is currently not mandated by Italian laws as an integral part of it.

This study shows the absence of differences in the outcome of interests between males and females. This is inconsistent with data from the literature, as anthropometric parameters have been found to alter participants’ performance; Contri *et al*.^31^ found that higher weight, height, BMI, and male sex provided better performance, achieving a greater percentage of adequate compression depth than other individuals.

It is of utmost importance to note that the medical school curriculum does not include a standard BLSD course. This affected the performance of the young physicians during the evaluation, both theoretically and practically. The mission and vision of the SIMED (Società Italiana di Medicina e Divulgazione Scientifica – *Italian Society of Medicine and Scientific Divulgation*) is to provide and integrate quality medical education, according to the latest scientific evidence, to all physicians with special emphasis on young professionals, with the implementation of mandatory courses for new physicians. Targeted interventions are effective in strengthening the performance of young physicians, ensuring that they are best equipped to deal with all clinical scenarios, especially emergency ones in dire times such as those faced during the recent pandemic. Simulation technologies are effective in improving the learning experience and satisfaction of healthcare workers while also showing improved outcomes in terms of knowledge, attitudes, and best practices acquired.^30,32–34^

Reports from different parts of the world have raised doubts about the CPR competencies of medical and other healthcare students.^28^ Lack of knowledge of cardiac arrest, CPR, and other life-saving maneuvers is not an issue pertaining solely to healthcare students but also for practicing nurses and physicians, whose knowledge of BLSD and ACLS has been shown to be suboptimal, if not poor, in different parts of the world. At present, there are few European countries that have made it mandatory by law, even for different levels of education.^35^

The lack of ability to perform BLSD by the physicians and the positive impact of the training course may be a useful way to define the importance of the training course in ensuring enhanced patients’ safety. Indeed, the achievement of approximately 92.5% of physicians able to perform effective and quality CPR after a specific BLSD training course demonstrates the importance and effectiveness of mandatory BLSD training for medical students.^23,28^ This highlights the importance of standardized and international training courses to increase the ability to perform BLSD, which should become mandatory for newly licensed physicians.

Further research in this area could be useful to explore the role of newly licensed physicians in the management of cardiac arrest in various scenarios, not forgetting the multifactorial nature of cardiac arrest. Moreover, this result was obtained using a nonparametric statistical test. Despite the loss of statistical power, the result was still statistically significant and more robust due to the elimination of assumptions about the data distribution.

This study presents several limitations. First, the practical test was carried out after the training course; future studies should show the ability to perform the maneuvers months later. Second, physicians who took part in the training course may have a lower ability to perform it. Third, weight, height, and BMI were not assessed, and correlation between anthropometric variables could have influenced participants’ performance.

## Conclusion

The BLSD course is definitely effective in improving participants’ practical ability to perform quality CPR on a mannequin. The results of this study suggest that further emphasis needs to be placed on the practical aspect of CPR training, and that newly licensed physicians should compulsorily participate in a BLSD course before starting their work after graduating from medical school.

Despite the reasonable sample size, the authors encourage and aim to replicate the results in larger samples, adding more data to the debate, which is of critical importance for the quality training that medical professionals need.

## Acknowledgements

This research received no external funding.

The study was conducted following the principles of the Helsinki Declaration and the Ethical principles in sports medicine. It was approved by the Società Italiana di Medicina e Divulgazione Scientifica (SIMED) research team. All research participants were volunteers, registered in SIMED networks. All SIMED members gave their consent to participate in the studies of the scientific society.

G.S. conceived and designed the study; G.S. and L.G. collected data; L.B. analyzed the data; G.S., P.S., M.I.S, A.B., N.F., and D.S. interpreted the results of the experiments; L.G., L.B., G.S., and N.F. drafted the first version of the manuscript. All authors edited and revised the manuscript. All authors have read and agreed to the published version of the manuscript.

The data presented in this study are available on request from the corresponding author.

### Conflicts of interest

There are no conflicts of interest.
